# Improved antitumor activity of TRAIL fusion protein via formation of self-assembling nanoparticle

**DOI:** 10.1038/srep41904

**Published:** 2017-02-22

**Authors:** Kaizong Huang, Ningjun Duan, Chunmei Zhang, Ran Mo, Zichun Hua

**Affiliations:** 1 State Key Laboratory of Pharmaceutical Biotechnology, School of Life Sciences and Affiliated Stomatological Hospital, Nanjing University, Nanjing, Jiangsu 210046, P.R. China; 2 State Key Laboratory of Natural Medicines, China Pharmaceutical University, Nanjing, Jiangsu 210009, P.R. China; 3 Nanjing Industrial Innovation Center for Pharmaceutical Biotechnology, Nanjing, Jiangsu 210019, P.R. China; 4 Changzhou High-Tech Research Institute of Nanjing University, Changzhou, Jiangsu 213164, P.R. China

## Abstract

Tumor necrosis factor-related apoptosis-inducing ligand (TRAIL) has been known as a promising agent for cancer therapy due to its specific apoptosis-inducing effect on tumor cells rather than most normal cells. However, systemically delivered TRAIL suffers from a rapid clearance from the body with an extremely short half-life. Thermally responsive elastin-like polypeptides (ELPs) are a promising class of temperature sensitive biopolymers based on the structural motif found in mammalian tropoelastin and retain the advantages of polymeric drug delivery systems. We therefore expressed RGD-TRAIL fused with ELP (RGD-TRAIL-ELP) in *E. coli*. Purification of RGD-TRAIL-ELP was achieved by the conveniently inverse transition cycling (ITC). The purified RGD-TRAIL-ELP without any chemical conjugation was able to self-assemble into nanoparticle under physiological condition. Non-reducing SDS-PAGE results showed that trimer content of RGD-TRAIL-ELP increased 3.4-fold than RGD-TRAIL. Flow cytometry confirmed that RGD-TRAIL-ELP 3-fold enhanced apoptosis-inducing capacity than RGD-TRAIL. Single intraperitoneal injection of the RGD-TRAIL-ELP nanoparticle induced nearly complete tumor regression in the COLO-205 tumor xenograft model. Histological observation confirmed that RGD-TRAIL-ELP induced significant tumor cell apoptosis without apparent liver toxicity. These findings suggested that a great potential application of the RGD-TRAIL-ELP nanoparticle system as a safe and efficient delivery strategy for cancer therapy.

The tumor necrosis factor (TNF) family comprises several ligands, such as the prototype TNF-α, the Fas ligand (FasL) and TNF-related apoptosis-inducing ligand (TRAIL), which trigger apoptosis in susceptible cells by activating respective cell-surface receptors^1^,^2^. However, TRAIL possesses some notable differences from TNF-α and FasL. TNF-α and FasL activate similar pathways using the same signaling proteins but present unacceptable side effect when administered systemically^3^. For example, TNF-α stimulates proliferation, survival, migration, and angiogenesis in most cancer cells, resulting in tumor promotion^4^; some study demonstrated that upregulation of FasL expression by tumor cells may enable the tumor cells to kill antitumor immune effector cells by activating lymphocytes express Fas^5^. At the other hand, TRAIL exhibits much stronger apoptotic activity than other TNF family members, kills tumor cells more effectively than normal cells, and is unlikely to initiate inflammatory cascades following systemic administration. These unique features of TRAIL have attracted considerable attention on TRAIL as a potential therapeutic to treat human cancers^6^,^7^,^8^,^9^. Despite its great advantages and potency in cancer treatment, TRAIL has a short biological half-life in body and suffers from a rapid kidney clearance when systemically administered^10^,^11^.

To address this dilemma, a variety of strategies aiming to increasing TRAIL particles have been employed to significantly improve its circulating time *in vivo*, because the size of therapeutic agents significantly affects their circulation time in the blood stream^12^. The diameter is inversely related to renal clearance. Some study demonstrated that particles with a diameter smaller than 5–6 nm are rapidly cleared by the kidney (blood half-life <600 min), while increasing in particle diameter can significantly enhance the half-life of these agents in the blood and body^12^. Previous, some study demonstrated that TRAIL was rapidly cleared from the systemic circulation and had a short biological half-life (20 min)^13^. Improving pharmacodynamics and enhancing anti-tumor activity could obtain reasonable supporting from adding TRAIL to larger particles, such using liposomes conjugated with TRAIL^14^,^15^ or adding TRAIL to human serum albumin (HSA) nanopartilce^16^. However, TRAIL that was modified with HSA nanoparticle must be prepared in organic solvents and scale-up preparation of the fusion protein was limited^16^,^17^. Some researchers showed that PEGylated TRAIL could preserve the biological activity of TRAIL and exhibited a remarkably improved pharmacokinetic profile. On the other hand, the PEGylated drug encountered the accelerated blood clearance (ABC) phenomenon and lose the long-circulating behaviors after repeated systemic injection^18^,^19^. Many attempts had been made to optimize the therapeutic efficacy by encapsulating TRAIL into liposomes or poly (lactic-co-glycolic acid) (PLGA), which could sustain the release of TRAIL from these carriers and reduce the frequency of systemic administration. Unfortunately, several problems associated with microsphere fabrication and agent release system occurred, such as exposure of the protein to an aqueous organic interface, freeze-drying, and physical instability in non-physiological microenvironment^20^,^21^,^22^. In addition, release of TRAIL from these carriers remains a challenge^23^.

Elastin-like polypeptides (ELPs) are biopolymers consisting of repeated peptide sequence VPGXG (derived from human tropoelastin^24^. ELPs can be encoded in the gene level, which allows a precise control over biopolymer composition and molecular weight and can be rapidly purified by inverse transition cycling (ITC)^25^. Furthermore, ELPs could self-assemble into nanoparticle when heated to relevant temperature^26^. As an attractive drug carrier, ELPs are biodegradable and nontoxic as well as capable of improving the systemic circulation and biodistribution^27^. These merits of ELPs provide a greatly promising application in drug delivery^25^,^28^. For example, ELP fusion protein for applications in diabetes treatment has been studied on phase II clinical trial.

We had demonstrated that RGD-TRAIL had a higher affinity with some cancer cells and showed enhanced therapeutic efficacy in suppressing tumor growth of the human colon carcinoma (COLO-205) tumor-bearing mice, compared to free TRAIL with the equivalent dose^29^. Nevertheless, RGD-TRAIL with small size also displayed short systemic circulation *in vivo*. Additionally, the existence of five cysteine residues and disulfide bond in RGD-TRAIL indicated that the soluble RGD-TRAIL was hardly achieved from *E. coli*.

Previously, our result had shown RGD-TRAIL fused with ELP (ELP [V_5_-60]) can be rapidly purified by ITC, improve apoptosis activity and self-assemble into microparticle. RGD-TRAIL fused with ELP (ELP [V_5_-60] would aggregate at 30 °C (5 μM)^30^. Accordingly, to optimize design, the RGD-TRAIL fused with relatively hydrophilic ELP (ELP [VH_4_-40]). Taking advantage of aggregative character of ELP, the polypeptide (RGD-TRAIL-ELP) was expected to be rapidly purified, formed more trimer and was able to self-assemble into nanoparticle under physiological condition (37 °C). Furthermore, due to increasing to diameter, once administrated to mice, RGD-TRAIL-ELP nanoparticle was suggested to improve the systemic circulation, generate more accumulation and elevate antitumor activity (Fig. 1).

Our result suggested RGD-TRAIL delivery system was better than free TRAIL *in vitro* and *vivo*^22^, therefore, we took RGD-TRAIL as control in following experiments.

## Results

### RGD-TRAIL-ELP expression and purification

**Figure 2:**
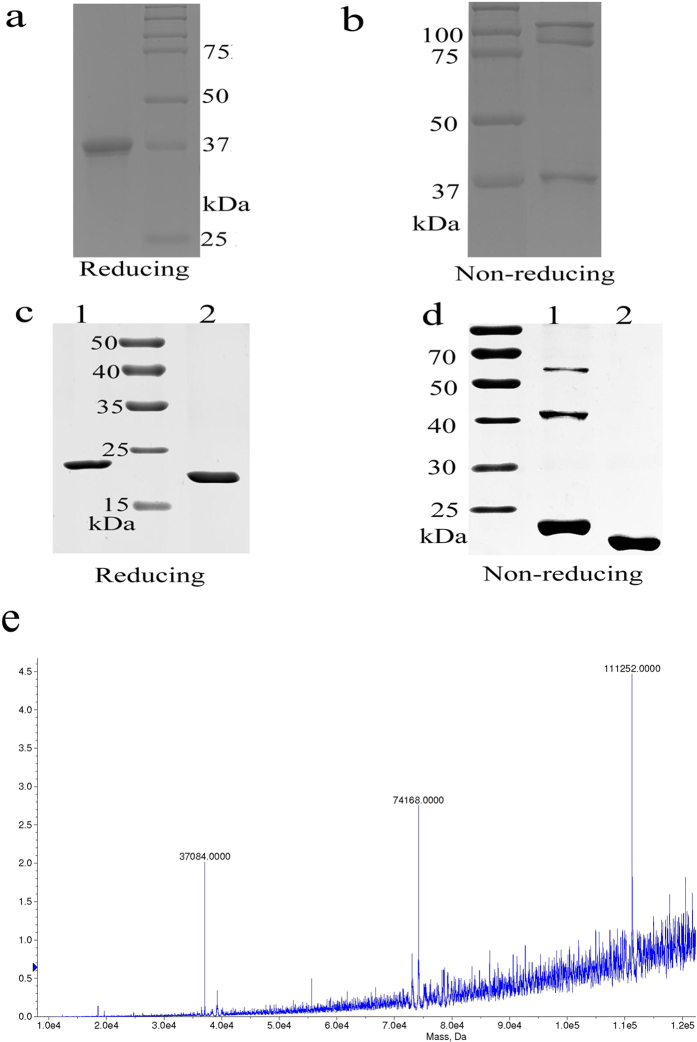
RGD-TRAIL-ELP were analyzed under nonreducing (−DTT, Fig. 2a) and reducing (+DTT, Fig. 2a) conditions by PAGE; RGD-TRAIL (lane 1) and ELP (lane 2) were analyzed under (+DTT, Fig. 2c) and reducing nonreducing (−DTT, Fig. 2d) conditions by PAGE; Maldi-TOF analyzed RGD-TRAIL-ELP (Fig. 2e).

The RGD-TRAIL-ELP recombinant proteins were successfully expressed in a soluble form and the final yield was about 20 mg/L. RGD-TRAIL-ELP was then analyzed by non-reducing and reducing SDS-PAGE. The reducing SDS-PAGE of RGD-TRAIL-ELP showed a single band of 37 kDa, consistent with the expected molecular weight (Fig. 2a). TNF family ligands, like TRAIL, can appear as monomer, dimer, and trimer forms. RGD-TRAIL-ELP also existed as a multimer form (Fig. 2b). Maldi-TOF (Fig. 2e) yielded a MW of 37087, 74168, 111252 Da, pointed to monomeric, dimeric and trimeric RGD-TRAIL-ELP. According to Fig. 2, it was estimated that 38% of RGD-TRAIL-ELP or 12% of RGD-TRAIL formed trimer respectively (Fig. 2b,d)^29^. Therefore, fusing ELPs chimeric polypeptide could contribute to form more multimers of TRAIL fusion protein.

### Characterization of the RGD-TRAIL-ELP nanoparticle

**Figure 3:**
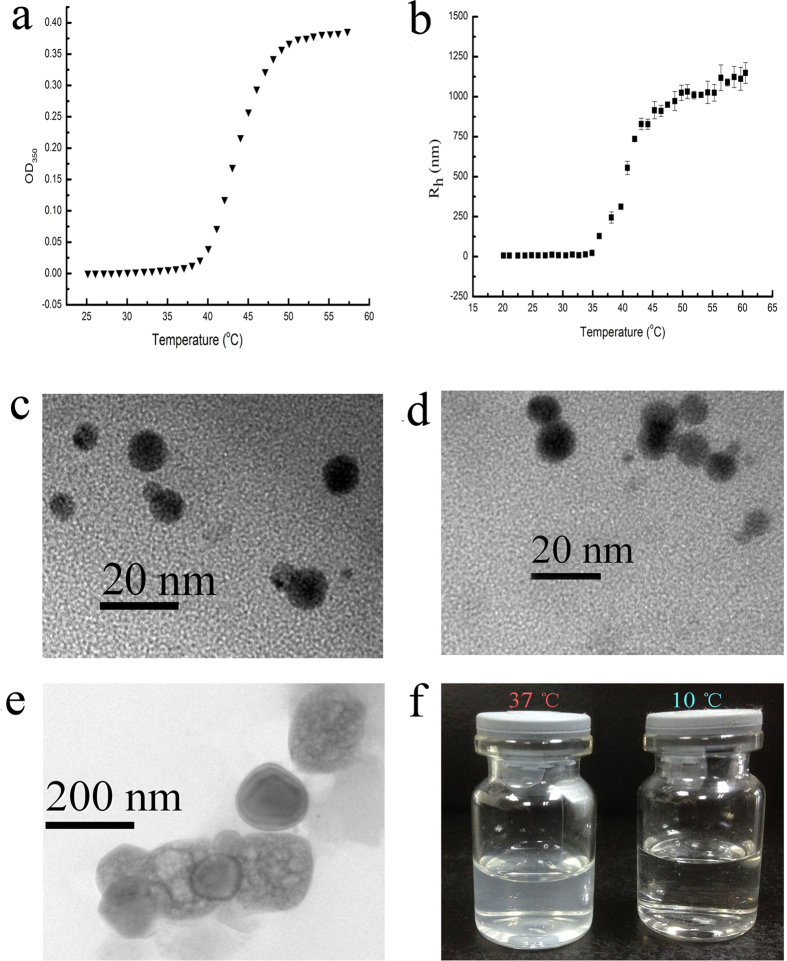
Characterization of RGD-TRAIL-ELP. Turbidity (**a**) and hydrodynamic diameter (**b**) profiles for RGD-TRAIL-ELP across range temperature at a concentration of 500 μg/ml in PBS; Freeze-fracture TEM images of RGD-TRAIL (**c**) and RGD-TRAIL-ELP (**d**, at 10 °C; **e**, at 37 °C); photographic image of RGD-TRAIL-ELP prepared at 10 and 37 °C (**f**).

As demonstrated in Fig. 3a, RGD-TRAIL-ELP began to aggregate near 40 °C. According to ref. 31, transition temperature was defined as the solution temperature at the maximum of the turbidity gradient, we estimated that transition temperature was 44 °C at concentration of 500 μg/ml^17^.

Figure 3a demonstrated that hydrodynamic diameter dramatically increased at 40 °C, which is consistent with the transition temperature result that significant aggregation was observed near 40 °C. The average diameter of RGD-TRAIL-ELP reached to ~190 nm at 37 °C (Fig. 3b and Supplementary Fig. S1). In sharp contrast, RGD-TRAIL itself only had the diameter around 8 nm regardless of the temperature (Fig. 3c). It is suggested that ELP motif greatly enhanced RGD-TRAIL’s propensity towards aggregation or assembly in response to the surrounding temperature. Additionally, the size of the RGD-TRAIL-ELP (37 °C) nanoparticle determined from the freeze fracture TEM (Fig. 3e) images was in agreement with that obtained from the DLS measurement.

### Biological activity

**Figure 4:**
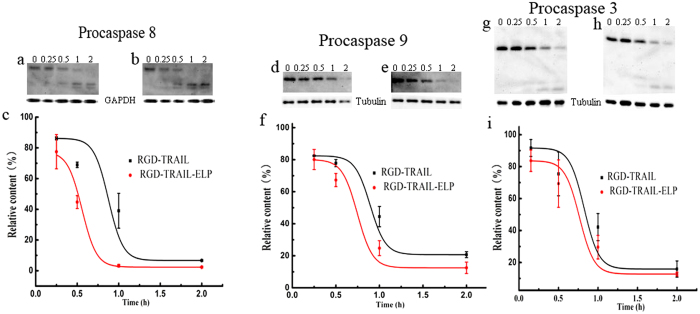
Western blot analysis of procaspase cleavage levels after COLO-205 cells were exposed to RGD-TRAIL and RGD-TRAIL-ELP over time (0, 0.25, 0.5, 1, 2 h). procaspase 8 (**a**, RGD-TRAIL; **b**, RGD-TRAIL-ELP); procaspase 9 (**d**, RGD-TRAIL; **e**, RGD-TRAIL-ELP); procaspase 3 (**g**, RGD-TRAIL; **h**, RGD-TRAIL-ELP). Densitometric analysis of procaspase cleavage levels, relative content was obtained from the densitometry that the given time over the zero time, procaspase 8 (**c**); procaspase 9 (**f**); procaspase 3 (**i**).

To compare the biological activity of RGD-TRAIL and RGD-TRAIL-ELP *in vitro*, we monitored caspase cleavage following treatments by two TRAIL variants because caspases were proportional to the potency of the TRAIL^5^. Treatment with RGD-TRAIL resulted in cleavage of procaspases 8, 9 and 3, but significant amounts of procaspases remained after 1 h of treatment. In comparison, more rapid cleavage of cellular procaspase was detected and most of procaspases disappeared by 2 h when after RGD-TRAIL-ELP treatment (Fig. 4). These data verified higher potency of RGD-TRAIL-ELP in apoptosis induction than RGD-TRAIL.

**Figure 5:**
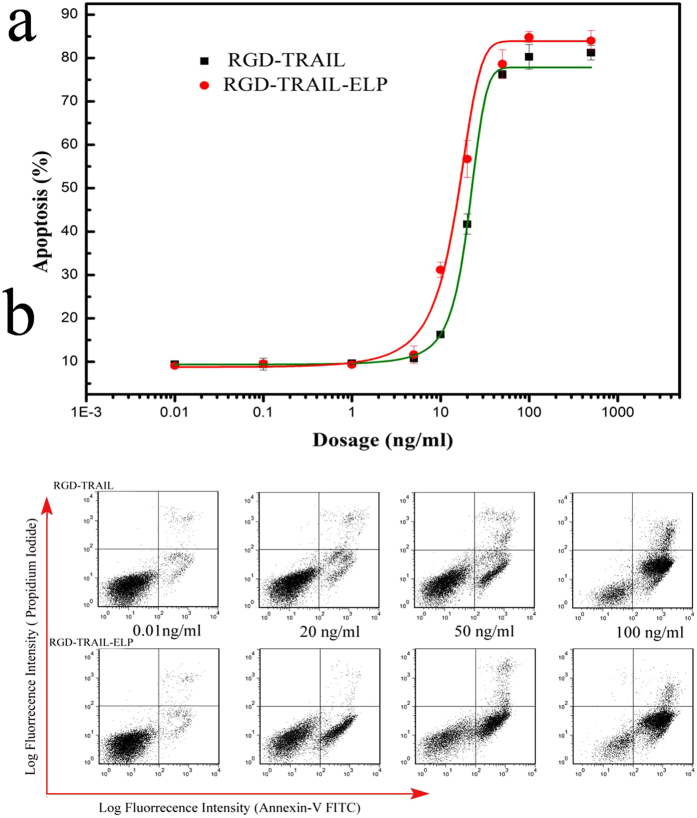
Dose-dependent apoptosis-inducing effect of RGD-TRAIL and RGD-TRAIL-ELP on COLO-205 cells (**a**); effect of RGD-TRAIL and RGD-TRAIL-ELP at different concentrations on COLO-205 cell death and apoptosis as determined by FACS using the Annexin V-FITC/PI staining (**b**).

We also investigated the biological activity of RGD-TRAIL-ELP. After treatment with serial dilutions of RGD-TRAIL and RGD-TRAIL-ELP, COLO-205 cells were stained with annexin V-FITC and PI, followed by flow cytometry analysis (Fig. 5). The half maximal effective concentration (EC50) for inducing 50% apoptosis of RGD-TRAIL and RGD-TRAIL-ELP was 1 and 0.35 nM (20.57 and 13.61 ng/ml), respectively, which suggest that RGD-TRAIL-ELP had a 3-fold enhanced apoptosis-inducing capacity than RGD-TRAIL. The elevated apoptosis-inducing capacity of RGD-TRAIL-ELP may be attributed to fact that the more homomultimer formed by RGD-TRAIL-ELP^8^, compared to less trimer formed by RGD-TRAIL that was reported in our previous work^29^. On the other hand, endothelial cells did not show apparent apoptosis by treating with RGD-TRAIL-ELP or RGD-TRAIL(500 ng/ml, Supplementary Fig. S2)

### Tumor accumulating capability

The tumor accumulation of different formulations in the COLO-205 tumor-bearing nude mice was evaluated after intravenous injection over time by near infrared spectroscopy. Representative images were shown in Supplementary Fig. S3. RGD-TRAIL exhibited rapid tumor-targeting capability in time-dependent property. As time lapsed, the fluorescence signals of Cy5.5-RGD-TRAIL waned at the tumor site and gradually increased in the liver. These results indicated that RGD-TRAIL presented a higher tumor-binding ability and might eventually degrade in the liver^29^,^32^.

In view of ELP, a high concentration of Cy5.5-ELP was primarily detected but was relatively faint in the tumor region over time. It is noteworthy that most of Cy5.5-ELP has been cleared from body followed by a further steady movement and was excreted by the renal pathway judged by the strong signals in bladder.

Cy5.5-RGD-TRAIL-ELP showed no remarkable intense fluorescence signal in the region of tumor within 2 h post-injection. As time extended, increased fluorescence signal of Cy5.5-RGD-TRAIL-ELP was observed at the tumor site compared with that at the normal tissues, suggesting superior ability of RGD-TRAIL-ELP to accumulate at tumor sites. Interestingly, after 24 h injection, the significant fluorescence accumulation was observed at the site of tumor and the scarce fluorescence signal was detected in other parts of body.

**Figure 6:**
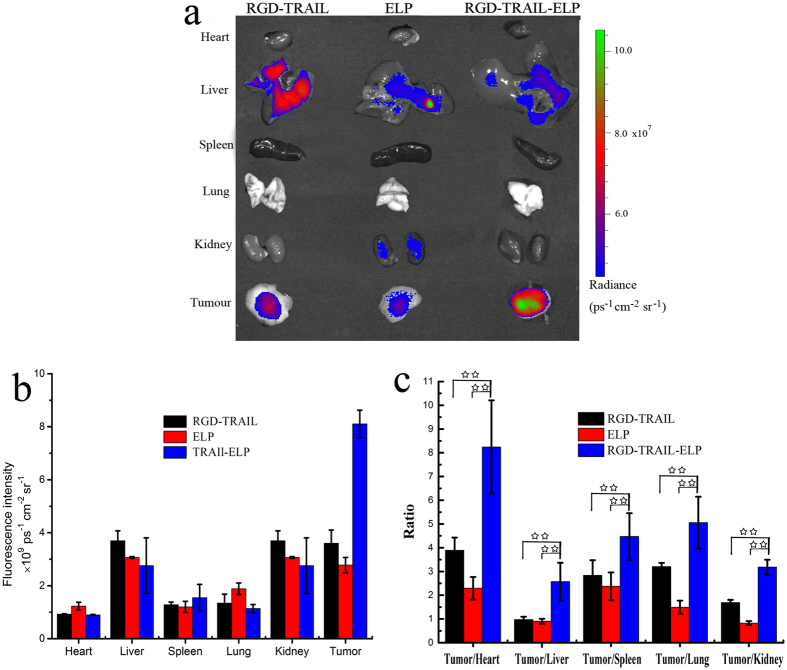
*Ex vivo* fluorescence imaging of the tumor and normal tissues harvested from the euthanized COLO-205 tumor-bearing nude mice at 24 h post injection of different Cy5.5-labeled formulations (**a**); quantification of fluorescent signal intensities of excised organs from mice at 24 h post injection. The data were recorded as total photons per centimeter squared per steradian (p/s/cm^2^/sr) (**b**); the selected target-to-accumulating background ratios of each studied group (**c**). ^**^P < 0.01.

At 24 h post-administration, the mice were immediately euthanized. The tumors and normal tissues were harvested for *ex vivo* imaging. As expected, for the RGD-TRAIL and ELP groups, only a small deposition of fluorescence was detected in the tumor, while a higher intensity of fluorescence signal was detected in the liver and kidney. RGD-TRAIL indeed displayed a relatively higher tumor-specificity than ELP control protein (Fig. 6). In the RGD-TRAIL-ELP group, a greater amount of fluorescence was enriched in the tumor, which was 2.5-fold higher than that in the RGD-TRAIL group; other tissues, such as heart, liver, spleen, lung and kidney, presented relatively weaker intensity, which further confirmed the tumor oriented accumulation of RGD-TRAIL-ELP. The selected target-to-background ratios were further determined by comparing the fluorescence intensity of Cy5.5 at the tumor to that at normal tissues in each studied group (Fig. 6c and Table 1). It is confirmed that the RGD-TRAIL-ELP nanoparticle presented a stronger capability of selective accumulation at the tumor tissue than either RGD-TRAIL or ELP.

To verify the accumulation of RGD-TRAIL or RGD-TRAIL-ELP at the tumor site by i.p. administration, tumor tissues were obtained four days after treatment. The distribution of RGD-TRAIL or RGD-TRAIL-ELP in the tumor tissue was analyzed using the immunofluorescence staining. In the RGD-TRAIL-ELP group, much stronger intensity of the fluorescence signal was detected, compared to another group (Supplementary Fig. S4). These results further indicated that the self-assembled RGD-TRAIL-ELP nanoparticle also showed more accumulation than the RGD-TRAIL in the tumor tissue by i.p. administration.

### *In vivo* pharmacokinetics

As shown in Supplementary Fig. S5 and summarized in Table 2, substantial differences were observed between the plasma pharmacokinetics of RGD-TRAIL and RGD-TRAIL-ELP. The half-life of RGD-TRAIL-ELP was 4.5 fold higher than that of RGD-TRAIL alone. The area under the curve from zero to infinity of RGD-TRAIL-ELP was 2.9 fold greater than that of RGD-TRAIL, and the plasma clearance of RGD-TRAIL-ELP was only 0.26 mL/min.

### *In vivo* antitumor efficacy

**Figure 7:**
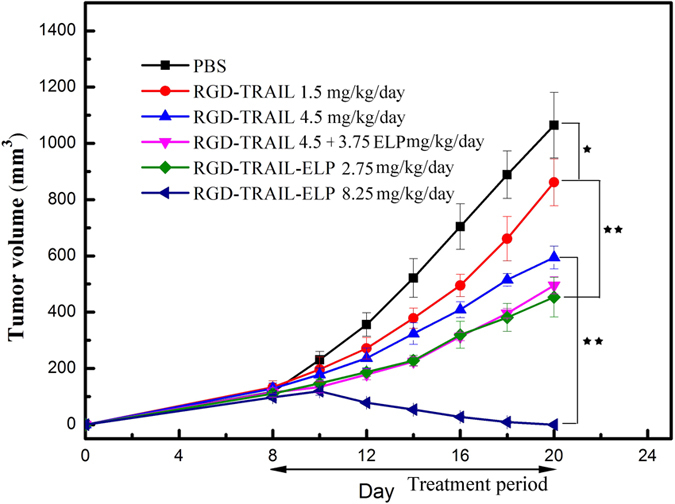
Change in the tumor size of the COLO-205tumor-bearing mice after treated with RGD-TRAIL or RGD-TRAIL-ELP. *P < 0.05, **P < 0.01.

The *in vivo* anti-tumor efficacy of RGD-TRAIL and RGD-TRAIL-ELP was evaluated in the COLO-205 tumor-bearing mice (Fig. 7 and Supplementary Fig. S6). Single administration of RGD-TRAIL and RGD-TRAIL-ELP were compared. Treatment with RGD-TRAIL at a dose of 1.5 mg/kg/day showed a slight response to the tumor compared with the PBS control. In contrast, treatment with the equimolar RGD-TRAIL-ELP (2.75 mg/kg/day) significantly inhibited the tumor growth. Furthermore, RGD-TRAIL-ELP at such dose achieved a higher tumor suppression effect than RGD-TRAIL at a high dose of 4.5 mg/kg/day. Co-delivery of RGD-TRAIL with free ELP showed unconspicuous antitumor effect than treatment with RGD-TRAIL at the same dose of 4.5 mg/kg/day. Notably, abolished tumors after injection day 10 was observed in the tumor-bearing mice treated with RGD-TRAIL-ELP at a higher dose of 8.25 mg/kg/day.

### Tissue staining and histological analysis

**Figure 8:**
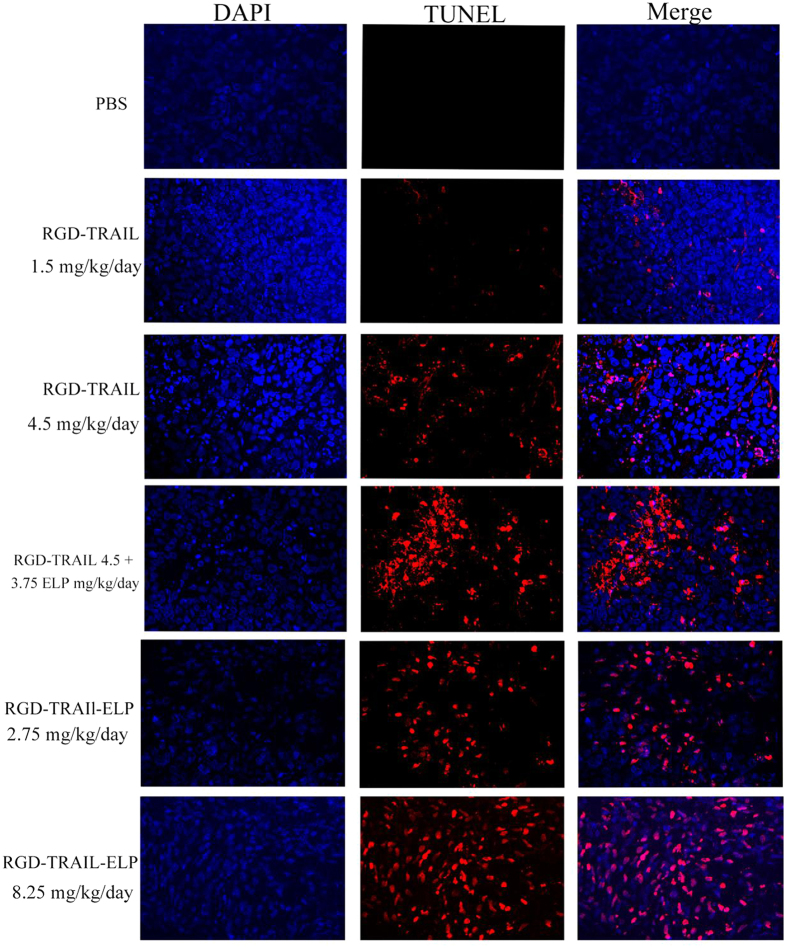
Confocal images of the tumor section obtained from the tumor-bearing mice after treatment with different formulations using the TUNEL staining (apoptotic and necrotic cells are shown in red) (×400).

An evaluation of apoptosis using the TUNEL assay revealed the clear presence of DNA fragmentation during apoptotic nuclei in the tumors of the mice after treated with RGD-TRAIL or RGD-TRAIL-ELP (Fig. 8). Greater amounts of tumor cell destruction were observed in the samples of RGD-TRAIL (4.5 mg/kg/day) or that plus free ELP, but apoptosis cells were rarely detected at lower dose (1.5 mg/kg/day) of the RGD-TRAIL group. Compared to the RGD-TRAIL groups, a larger amount of apoptotic cells were observed in the tumor tissues from the mice after treated with RGD-TRAIL-ELP, which resulted probably from a combination between the enhanced delivery efficiency of the nanoparticle and the bioactivity of RGD-TRAIL-ELP.

To confirm the *in vivo* therapeutic effects of RGD-TRAIL-ELP, histological detection was also performed (Supplementary Fig. S7). Similar to the TUNEL staining result, treatment with RGD-TRAIL-ELP markedly increased the number of apoptotic cells, which further supported the superior antitumor efficacy of RGD-TRAIL-ELP *in vivo*.

The liver cytotoxicities of RGD-TRAIL and RGD-TRAIL-ELP were also evaluated using histological detection, respectively (Supplementary Fig. S8). RGD-TRAIL and RGD-TRAIL-ELP did not show any noticeable toxicity toward the murine liver cells. No significant variation in the body weight and parameter of toxicity in the kidneys during the treatment of both RGD-TRAIL and RGD-TRAIL-ELP also suggested the absence of serious toxic side effects (Supplementary Figs S9 and S10).

## Discussion

Up to now, many recombinant versions of TRAIL had been purified from *E. coli*. Unfortunately, most of recombinant TRAILs were produced as inclusion bodies in *E. coli*^33^,^34^. Inclusion bodies should be solubilized and refolded, which would be restricted in industrial preparation. One strategy to overcome this issue is to fuse peptide tags to TRAIL. For example, TRAIL fused with leucine zipper^35^, polyhistidine-tag^36^ or immunoglobulin Fc domain^37^ could improve its solubility and bioactivity, but the purification process is indispensably performed by complicated affinity chromatography that required time-, cost- and labor-consuming efforts. To improve the biological half-life of TRAIL, covalent conjugating TRAIL with PEG was utilized^23^,^38^,^39^,^40^. However, the possibility of introducing more issues by adding a long ‘tail’ to the monomer would bring about some issues, such as affecting trimerization, receptor-binding or molecular flexibility of TRAIL^41^, it was reported that the half maximal inhibitory concentration (IC50) of the PEGylated TRAIL was 4.2-fold higher than that of TRAIL alone^42^. Moreover, these carriers are hard to modulate property and the purification and linking TRAIL cannot be realized in just one step^43^.

Based on our preparation techniques, we expressed soluble RGD-TRAIL-ELP fusion protein, which could be easily and rapidly purified from *E. coil* by ITC. The ITC purification method is an economical and convenient procedure, which is also easy to scale-up^44^. Additionally, RGD-TRAIL-ELP can significantly improve apoptosis-inducing bioactivity by forming more homotrimers. More importantly, the fusion polypeptide can self-assemble into nanoparticle under physiological condition. Such integration of purification and self-assembly into a uniform nanoparticle in one step can efficiently avoid the structural disruption of TRAIL and facilitate the formation of stable TRAIL multimer.

As a genetically-encoded amphiphilic polypeptide, ELP or its fusion protein can self-assemble into nanoparticle for drug delivery^45^. Below the transition temperature, ELP exists as a soluble monomer; when the temperature is raised above the transition temperature, the selective dehydration of ELP would become hydrophobic and therefore drive the self-assembly of ELP into a higher ordered hydrophobic core^46^,^47^. Previously, we described RGD-TRAIL delivery system for cancer therapy^22^. In the present study, we found that RGD-TRAIL fused to the ELP chimeric polypeptide displayed self-assembled property at 37 °C, and could contribute to form more multimers. Western blot also indicated that RGD-TRAIL-ELP triggered more efficient cleavage of caspases.

Most TRAILs are rapidly excreted by kidney, which results in a very short half-life and detracts the pharmaceutical application of TRAIL^23^. To address this issue, we enlarged the RGD-TRAIL size by taking advantage of the self-assembly property of ELPs, since the particle size is an important parameter impacting the fate of systemic delivery^48^. The self-assembly process of RGD-TRAIL-ELP at the body temperature was demonstrated by both the DLS measurement and the FF-TEM images. The RGD-TRAIL-ELP nanoparticle had a longer retention time in the blood circulation, reduced the clearance rate, and showed preference to accumulate at the tumor sites, compared with the RGD-TRAIL. Furthermore, the *in vivo* antitumor activity studies confirmed that the RGD-TRAIL-ELP nanoparticle produced an expected higher therapeutic efficacy than the RGD-TRAIL. This effect was further substantiated by both TUNEL and H&E assays. More importantly, RGD-TRAIL-ELP induced nearly complete tumor regression without acute liver toxicity and notable change in the body weights after treating the tumor-bearing mice at a dose of 8.25 mg/kg/day.

In summary, we had successfully expressed and rapidly purified RGD-TRAIL-ELP by ITC, RGD-TRAIL-ELP could form more multimers and presented an enhanced apoptosis-inducing capability. Noteworthiness, RGD-TRAIL-ELP could self-assemble into nanoparticle under physiological conditions (pH 7.4, 37 °C), the size of which could be adjusted by the environmental temperature. The RGD-TRAIL-ELP nanoparticle system accomplished a much better bioavailability and significantly improved antitumor effect than the RGD-TRAIL solution *in vivo*. In addition, no evident sign of toxic effect on the liver was observed during the RGD-TRAIL-ELP treatment. Taken together, the admirable properties of RGD-TRAIL-ELP could offer a safe and attractive strategy for the application of the protein-based delivery system to treat cancer.

## Methods

### Statement

All experiments in current study were performed in accordance with relevant guidelines and regulations.

### Animals

Female nude mice (5–6 weeks old, ~20 g) were obtained from Academy of Chinese Military Medical Sciences. This experiment was approved by the Animal Ethics Committees of the Institute of Materia Medica, Chinese Academy of Medical Sciences and Nanjing University. Animals were maintained in a pathogen-free environment (23 ± 2 °C and 55 ± 5% humidity) through a 12 h light ~12 h dark cycle with food and water supplied across the experimental period. The animals were housed and cared for in accordance with the guidelines established by the National Science Council of Republic of China.

### ELP cloning, expression and purification

The oligonucleotides ELP [VH_4_-5] were synthesized by Genscrips (Nanjing China). ELP [VH_4_-40] was constructed using plasmid reconstruction recursive directional ligation^49^. ELP [VH_4_]-40 contained 8 monomeric ELP pentapeptides ELP[VH_4_-5], which has a repeat unit composed of 5 pentapeptides with the guest residues Val and His in 1:4 ratio^50^. ELP [VH_4_-40] was cloned into the modified pET-23a-RGD-TRAIL (*Bam*HI and *Xho*I), the vector was named as pET-23a-RGD-TRAIL-ELP and correct clones were identified by DNA sequencing. RGD-TRAIL-ELP was expressed and purified by employing ITC^51^. In brief, one round of ITC is described as follows: (1) the RGD-TRAIL-ELP solution with sodium chloride was supplemented to a final concentration of ~1 M; (2) the solution was heated to the temperature of 37 °C to initiate the inverse phase transition; (3) centrifugation at 12,000 rpm to collect RGD-TRAIL-ELP at 37 °C; (4) the RGD-TRAIL-ELP pellet was resuspended in cold PBS at 4 °C; (5) the insoluble debris was removed by centrifugation at 13000 rpm 4 °C. The purified protein was achieved by three rounds of ITC and passed through a 10 mL of bed high-capacity endotoxin removal resin (Thermo Scientific).

### Protein analysis

Protein electrophoresis of purified TRAIL- ELP was performed under reducing (+DTT) and non-reducing (−DTT) conditions using Coomassie blue staining^29^. Maldi-TOF was performed to further analysis molecular weight of RGD-TRAIL-ELP.

### Characterization of nanoparticle

The transition temperature of RGD-TRAIL-ELP was calculated by recording the optical density at 350 nm as a function of temperature (1 °C/min) on a temperature controlled spectropolarimete J-815 (Jasco: Tokyo, Japan) equipped with a multicell thermoelectric temperature controller between 25 and 60 °C.

Dynamic light scattering (DLS) measurement was used to determine the particle radius of the RGD-TRAIL-ELP nanoparticle. Before measurement, RGD-TRAIL-ELP was filtered through a membrane filter with 0.1 μm size pores at 4 °C and was adjust to 500 μg/mL in phosphate buffered solution (PBS). Average size and size distribution of the RGD-TRAIL-ELP nanoparticles were then characterized using Dynamic Light Scattering (DLS; DynaPro Plate Reader, Wyatt Technology) over a range of temperature. The light scattering data were analyzed using a regularization fit and the Rayleigh spheres algorithm. Aberrant peaks were only eliminated if they accounted for less than 2% of the sample by mass.

The transmission electron microscope (TEM) characterization on the morphology of the RGD-TRAIL-ELP nanoparticle was performed by freeze fracture replication according to standard techniques^24^. Briefly, samples were heated to the temperature of 37 °C and rapidly cooled using the liquid nitrogen-cooled propane. The obtained samples were fractured by using a Leica EM VCT100 and the faces were coated with platinum at an angle of 30°. This process formed a replica of the fractured surface that was further cleaned with nitric acid prior to imaging^28^. The surface morphology of the RGD-TRAIL-ELP nanoparticle was via transmission electron microscope (JEM2100EX electron microscope, JEOL, Japan).

### Apoptosis assessment by Annexin V-FITC/propidume iodide (PI) staining and Western blot

COLO 205 cells were cultured in RPMI 1640, supplemented with 10% fetal bovine serum at 37 °C and 5% CO_2_ atmosphere.

To elucidate distinction in the activity between RGD-TRAIL and RGD-TRAIL-ELP, COLO-205 cells were exposed to 100 ng/ml RGD-TRAIL or RGD-TRAIL-ELP, and harvested at prearranged time interval. Procaspases 8, 9 and 3 were analyzed and compared by Western blot^8^.

To further determine the apoptotic activities of RGD-TRAIL and RGD-TRAIL-ELP, COLO 205 cells (10^6^ cells) were treated with RGD-TRAIL-ELP and RGD-TRAIL at range concentrations. The cells were harvested and washed with PBS thrice. Cell apoptosis and necrosis were analyzed using the Annexin V-FITC/PI staining as described previously^29^.

### Synthesis of Cy5.5-labeled recombinant protein

Cy5.5-labeled RGD-TRAIL (Cy5.5-RGD-TRAIL) and RGD-TRAIL-ELP (Cy5.5-RGD-TRAIL-ELP) were synthesized by reacting with the Cy5.5–NHS ester. For ELP, 5 lysine residues that were conjugated with Cy5.5 were introduced into the ELP sequence. Cy5.5-labeled recombinant protein was prepared as described previously in ref. 52. In brief, Cy5.5, SE (1 mg, 8.86 μM) was dissolved in 100 μL of dmethyl sulphoxide (DMSO). RGD-TRAIL, ELP and RGD-TRAIL-ELP were dissolved in 500 μL of 50 mM NaHCO_3_ (pH 8.5) and then mixed with Cy5.5, SE ([Cy5.5, SE]/[-NH2] = 5). After overnight incubation at 4 °C in dark, RGD-TRAIL-ELP and RGD-TRAIL were purified by dialysis against water in dark for 48 h at room temperature and the water was exchanged several times. Afterward, the solution was ultrafiltrated and concentrated using a centrifugal filter. The fluorescence spectra of the obtained Cy5.5-RGD-TRAIL and Cy5.5-RGD-TRAIL-ELP were measured using a fluorescence spectrophotometer (Shimadzu, Japan).

### *In vivo* real-time imaging

Female athymic nude mice bearing a subcutaneously COLO-205 tumor (approximately 350~500 mm^3^) were intravenously injected with Cy5.5-labeled RGD-TRAIL and Cy5.5-RGD-TRAIL-ELP at a dosage of 5 mg/kg (100 μg). *In vivo* fluorescence imaging was performed by scanning the mouse abdomen at the predetermined time interval (2, 4, 8, 24 h) using IVIS Lumina XR system (Caliper, Life science) with Cy5.5 excitation (640 nm) and emission (672 nm) filter sets. At 24 h post injection, the mice were sacrificed. Their major organs and tumor were harvested. Each organ and tumor were rinsed with saline three times and put into the board. To analyze the organ distribution, all data are calculated using the region-of-interest (ROI) function of analysis workstation software and data were given as mean ± S.D. for a group of three animals. A quantification of fluorescence signals was performed as total photons per centimeter squared per steradian (p/s/cm^2^/sr) per each organs and tumor.

### *In vivo* pharmacokinetics

The pharmacokinetic profiles of RGD-TRAIL and RGD-TRAIL-ELP were examined by intravenous administration. Plasma samples were obtained at different time points by centrifugation and stored at −20 °C until required for assay. The RGD-TRAIL and RGD-TRAIL-ELP concentration in serum were determined by using ELISA kits according to manufacturer’s instructions. Kinetica4.4 software was used to calculate pharmacokinetic parameters.

### *In vivo* antitumor efficacy

Evaluation of antitumor activity with a xenografted mice model was carried out as described previously. Briefly, 2.5 × 10^6^ of COLO-205 cells were resuspended in 100 μL of PBS, and subcutaneously implanted into the right flank region of the female BALB/c nu/nu mice. Treatment was initiated when the tumors reached a mean volume of ~100 mm^3^ (Eight days after tumor implantation). Before administration, the RGD-TRAIL-ELP was heated to the temperature of 37 °C to form nanoparticles. 1.5 or 4.5 mg/kg/day RGD-TRAIL or RGD-TRAIL-ELP (2.75 mg/kg/day or 8.25 mg/kg/day) were administered by i.p. injection for 14 days. Free ELP (3.75 mg/kg/day) plus RGD-TRAIL (4.5 mg/kg/day) was also injected by i.p. Tumor volumes were monitored every other day. Six groups were defined (n = 5). Mice were cared for in accordance with the guide for the care and use of laboratory animals. Tumors were measured with a caliper, and the tumor volume was calculated as width^2^ × length × 0.5.

### Tissue staining and histological analysis

The subcutaneous dorsa of female BALB/c nu/nu mice (6 weeks old) were inoculated with 2.5 × 10^6^ COLO-205 cells. To test targeting study by i.p. injection mice were randomly divided into six groups (n = 3) when the tumors reached ~100 mm^2^, and was administered i.p. Four days after treatment, tumor and liver were harvested, fixed in 4% paraformaldehyde, and embedded in paraffin and sectioned (5-μm thick). The tumor and liver sections were stained with hematoxylin and eosin (H&E), and observed by an optical microscope (Eclipse, E100, Nikon, Japan). Apoptotic cells in tumor tissues were assessed with the DAPI staining and terminal deoxynucleotidyl transferasemediated nick end labeling (TUNEL) assays with commercial apoptosis detection kit (Vazyme Corp., Nanjing). Samples were visualized by a confocal microscope (Nikon confocal microscope, C2+, Nikon, Tokyo).

For immunofluorescence, tumor tissue sections were incubated with fluorescein isothiocyanate (FITC)-conjugated secondary antibody (diluted 1:500 in PBS) for 60 min at room temperature. The tumor sections were then washed with PBS, incubated with DAPI (to stain nuclei), slide-mounted, and sealed. Localization of TRAIL (Rabbit mAb, Cell Signaling Technology) in tumor tissue was visualized using the confocal microscope^53^.

### Data analysis

All experiments were performed three times and data was expressed as the mean ± s.d. (n = 3). The significance of difference was determined using the Student’s t-test. P-values of <0.05 and <0.01 were considered as statistical significance and extreme significance, respectively.

## Additional Information

**How to cite this article:** Huang, K. *et al*. Improved antitumor activity of TRAIL fusion protein via formation of self-assembling nanoparticle. *Sci. Rep.* **7**, 41904; doi: 10.1038/srep41904 (2017).

**Publisher's note:** Springer Nature remains neutral with regard to jurisdictional claims in published maps and institutional affiliations.

## Supplementary Material

Supplementary Information

## Figures and Tables

**Figure 1:**
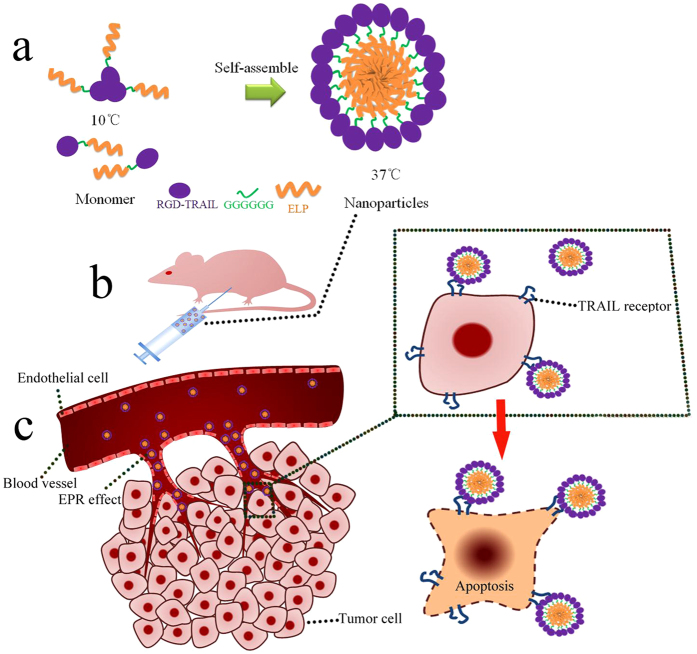
Schematic illustration of the RGD-TRAIL-ELP nanoparticle for tumor-targeted delivery. Before administration, the RGD-TRAIL-ELP was heated to the temperature of 37 °C to form nanoparticles (**a**); RGD-TRAIL-ELP was administered by i.p (**b**) and deposited in tumor site (**c**).

**Table 1:** Biodistribution of organs after 24 hours.

Tumor	Liver	Lung	Heart	Spleen	Kindey
44%	18%	8%	6%	10%	14%

**Table 2:** Comparing of pharmacokinetic parameter after adminstration RGD-TRAIL or RGD-TRAIL-ELP to rat at 100 μg (0.5 mg/kg) i.v, n = 4.

Pharmacokinetic parameter	RGD-TRAIL	RGD-TRAIL-ELP
ACU_inf_^a^ (ng·min/ml)	139623.75 ± 26170.08	402790 ± 41932.57
T_1/2_^b^ (min)	50.87 ± 9.14	230.05 ± 11.42
CL^c^ (ml/min)	0.94 ± 0.15	0.26 ± 0.026

^a^ACU_inf_: Area under the curve from zero to infinity.

^b^T_1/2_: Half-life.

^c^CL: Clearance.
