# Supplementary material for: Improved antitumor activity of TRAIL fusion protein via formation of self-assembling nanoparticle
Source: Sci Rep. 2017 Feb 22;7:41904. doi: 10.1038/srep41904 (PMC5320504; doi:10.1038/srep41904)
Supplement: Supplementary Information [file srep41904-s1.doc]

**Supplementary Information**

**Improved antitumor activity of TRAIL fusion protein via formation of self-assembling nanoparticle**

Kaizong Huang, Ningjun Duan, Chunmei Zhang, Ran Mo, Zichun Hua

**
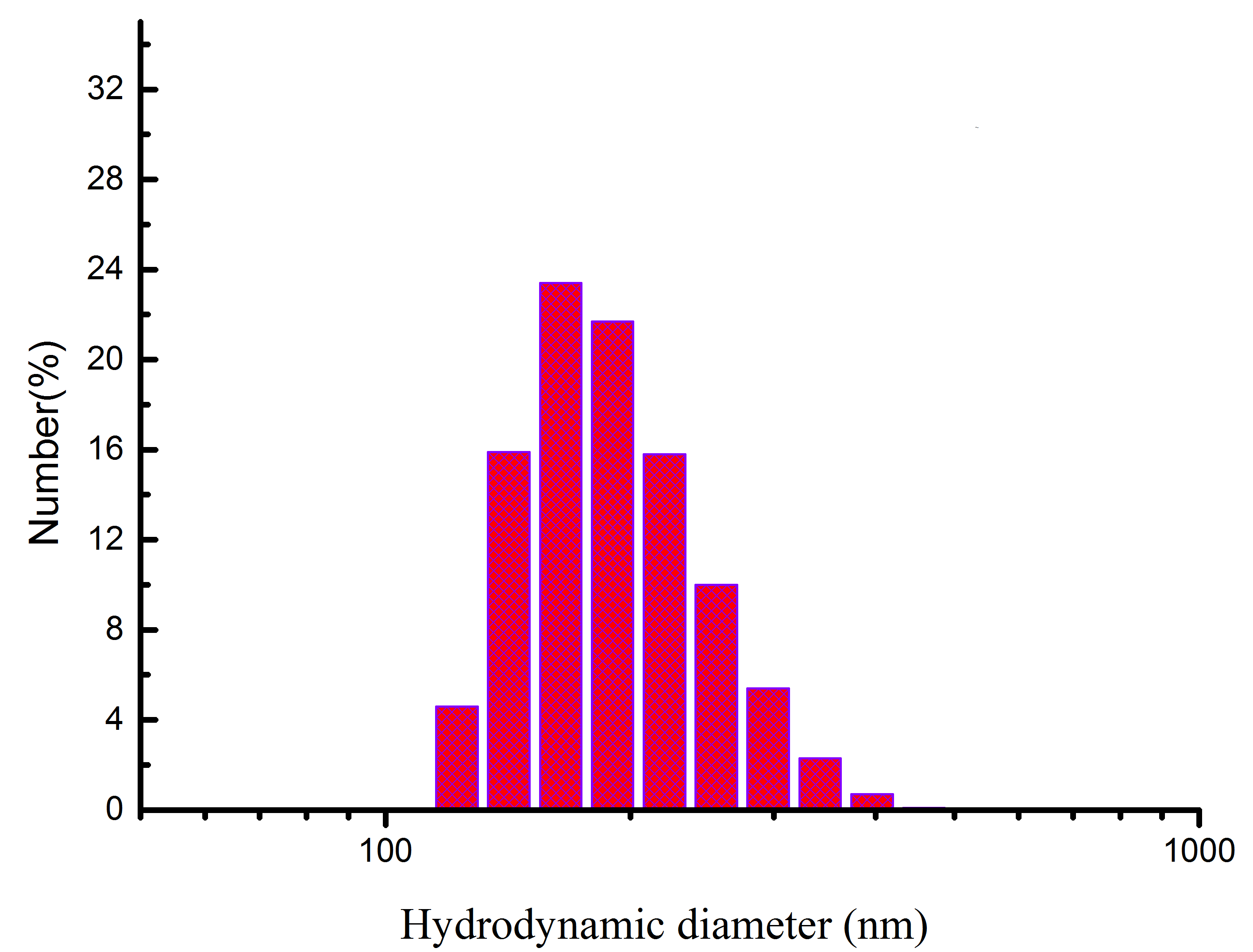
**

**Supplementary Fig. 1** DLS analyzed RGD-TRAIL-ELP at 37^o^C


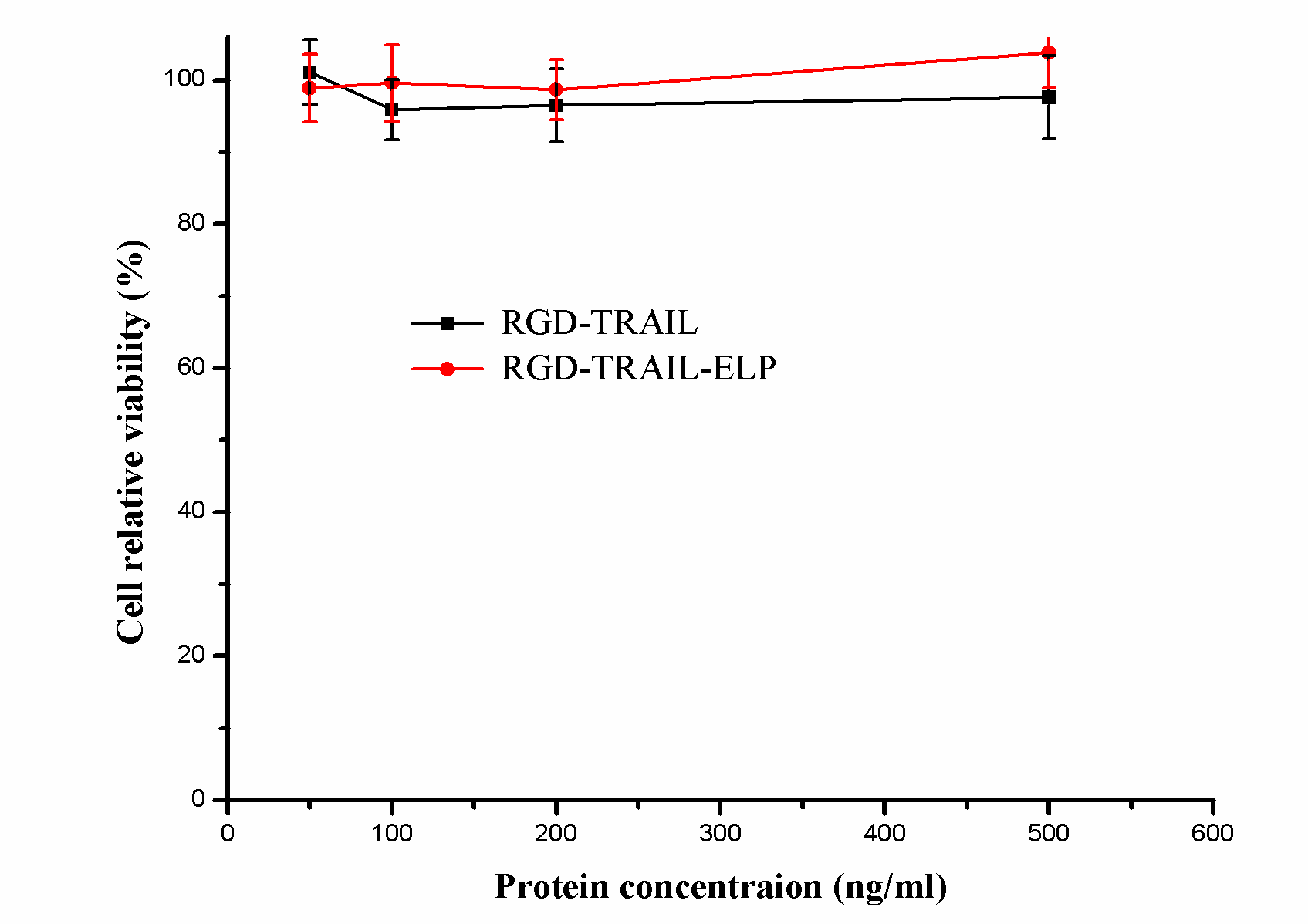


**Supplementary Fig. 2** RGD-TRAIL-ELP or RGD-TRAIL treated endothelial cell, cell viability was analyzed by MTT, values reported are the means ± SD for triplicate samples.


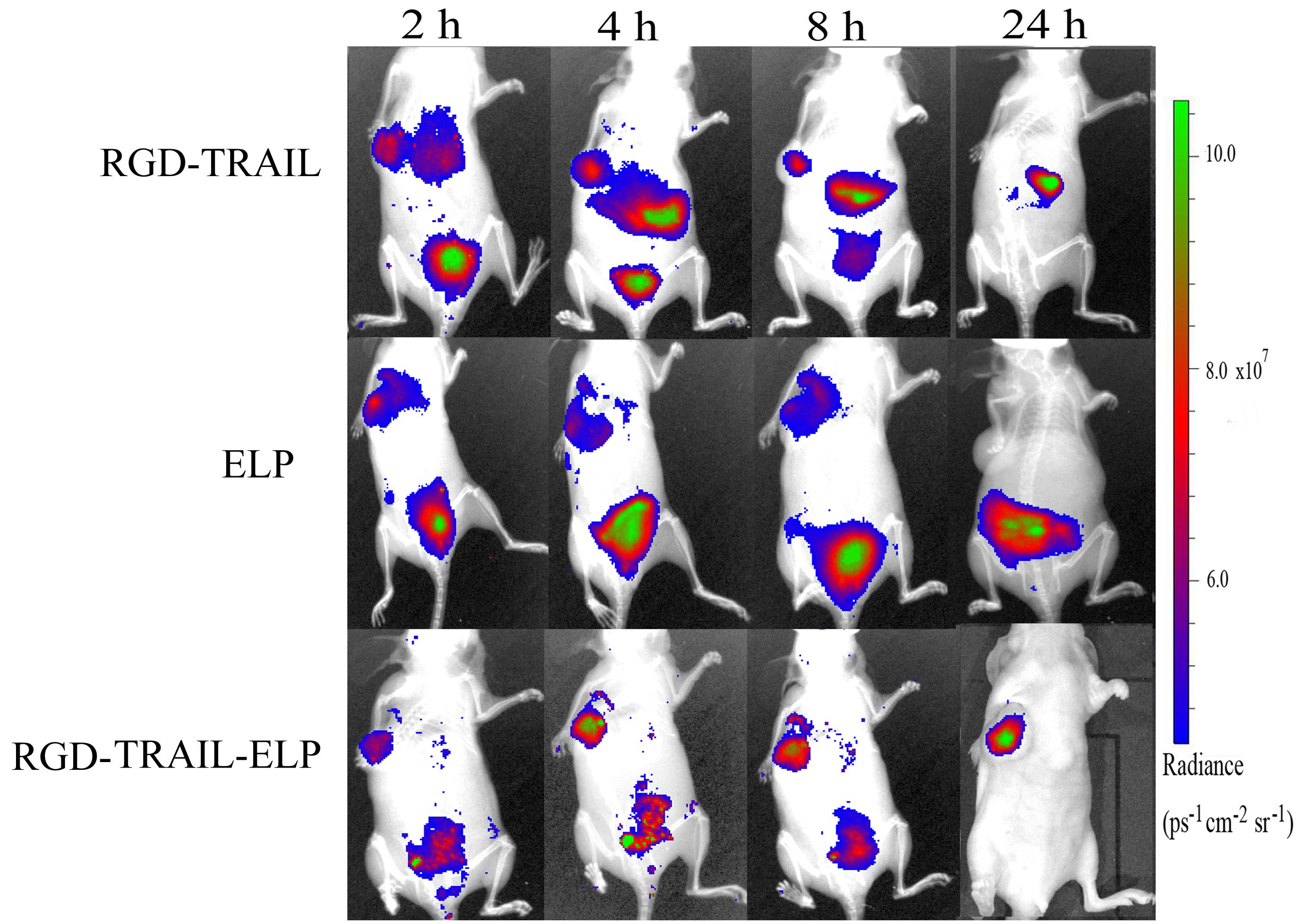


**Supplementary Fig. 3** In vivo fluorescence imaging of the COLO-25 tumor-bearing nude mice following intravenous injection with different Cy5.5-labeled formulations over time


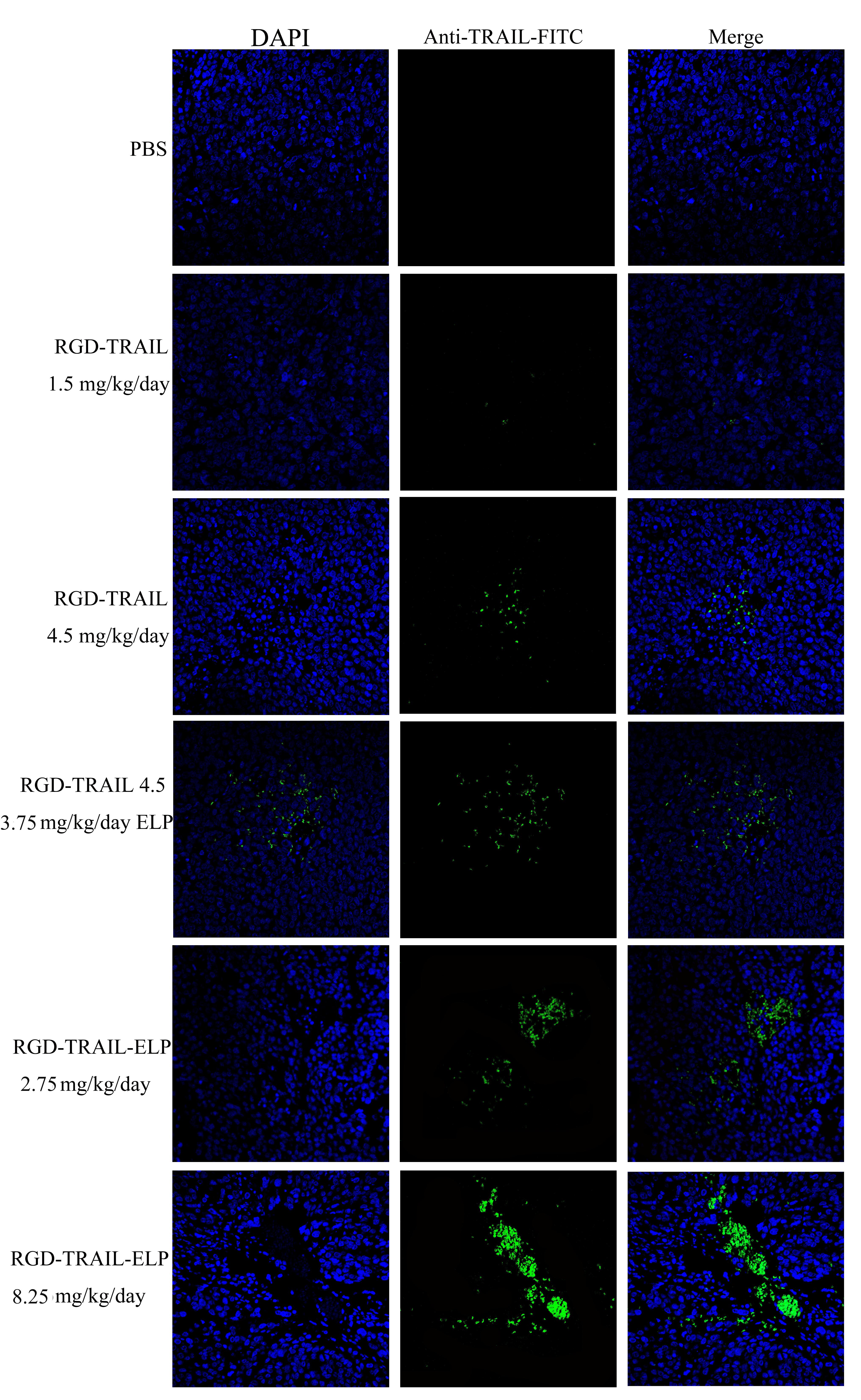


**Supplementary Fig. 4** Immunofluorescence analyze after RGD-TRAIL and RGD-TRAIL-ELP intraperitoneal administration in tumor section.


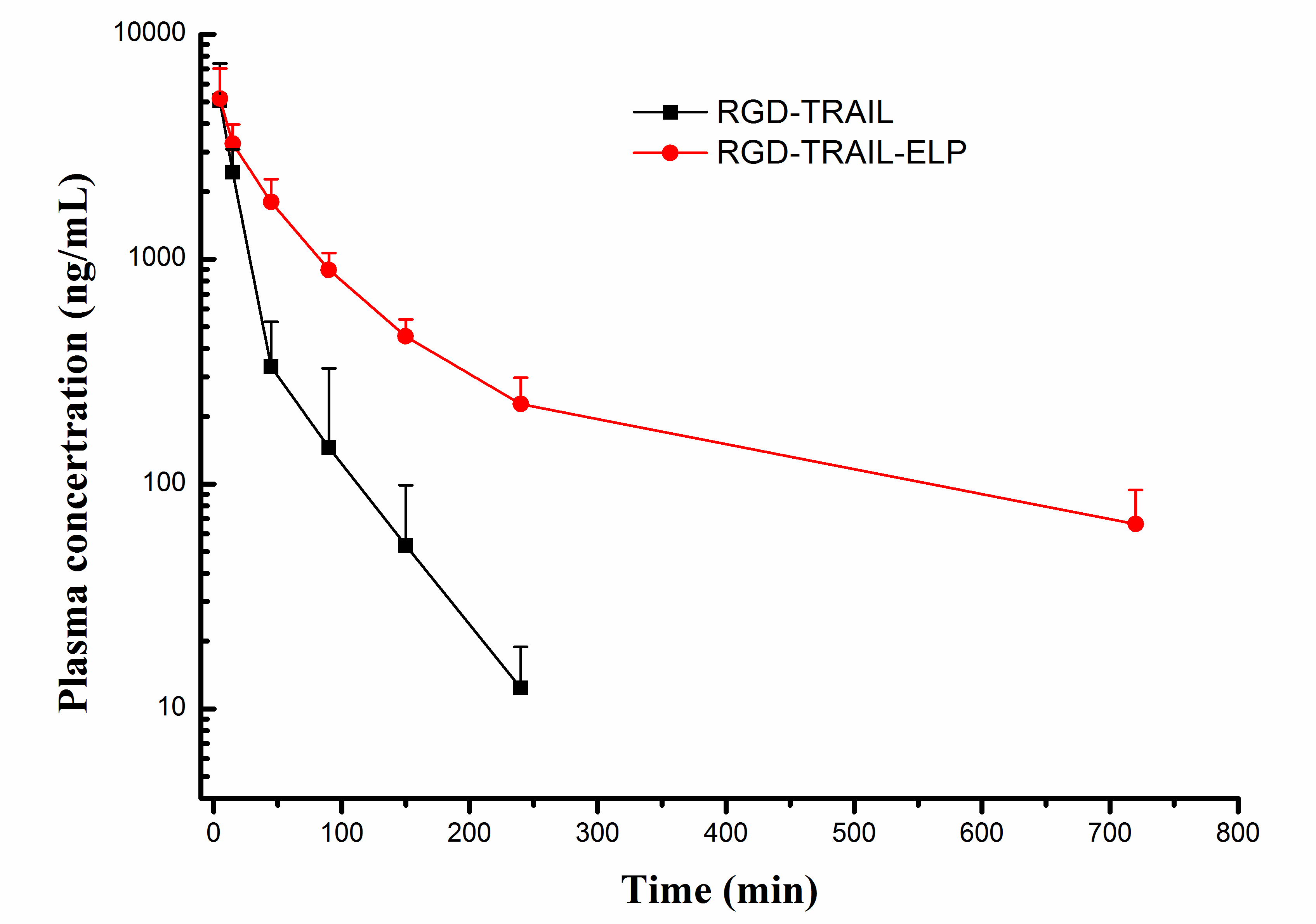


**Supplementary Fig. 5** Pharmacokinetic profiles of RGD-TRAIL and RGD-TRAIL-ELP. Sprague-Dawley (SD, ～200g) rats were administered an i.v. injection of RGD-TRAIL or RGD-TRAIL-ELP (100 μg per rat), and their concentrations in blood were determined by ELISA. Results are expressed as mean±S.D. from 4 different rats (n = 4).


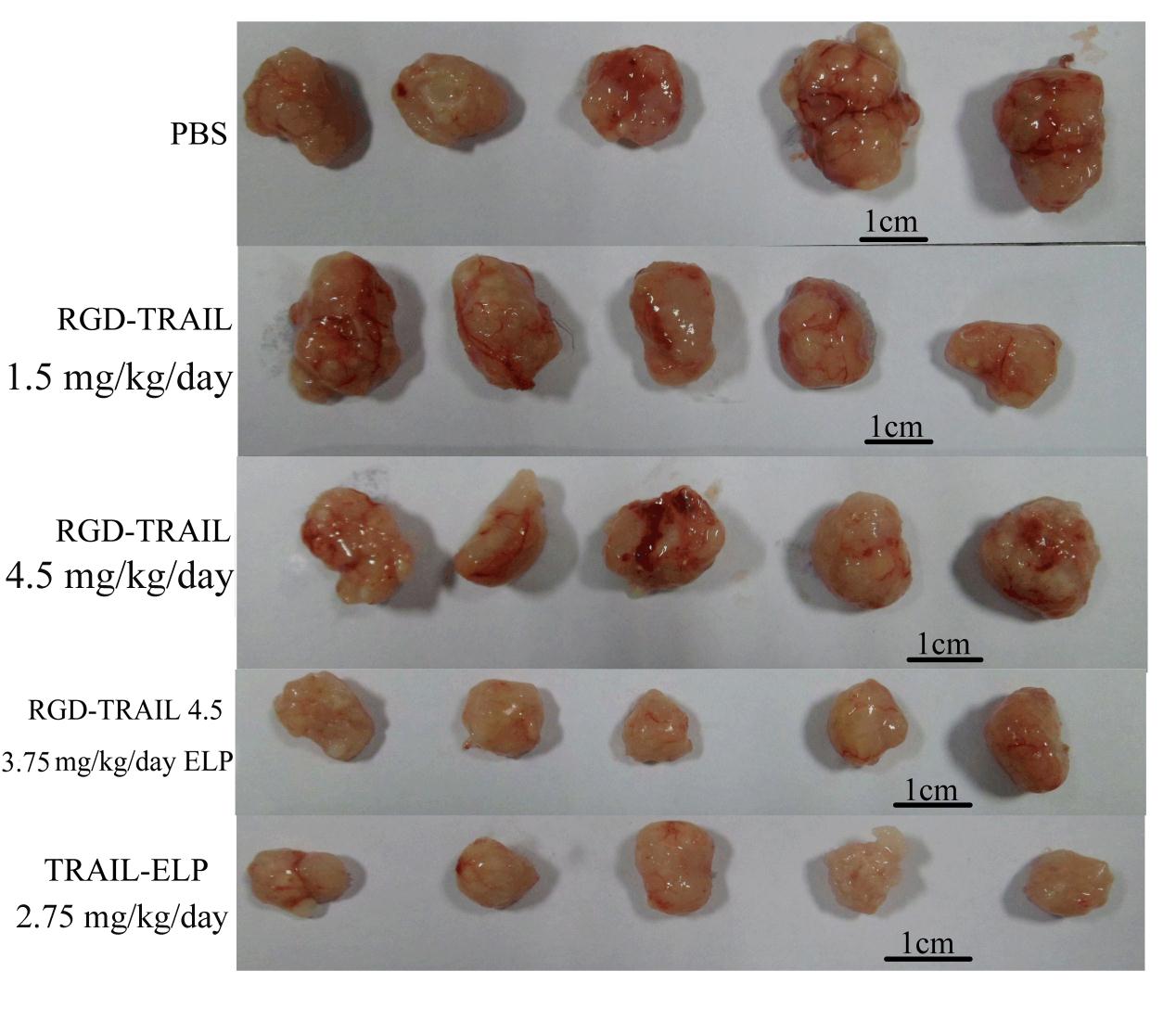


**Supplementary Fig. 6** Animals were sacrificed 14 days after withdrawing administration and tumors were harvested and imaged.


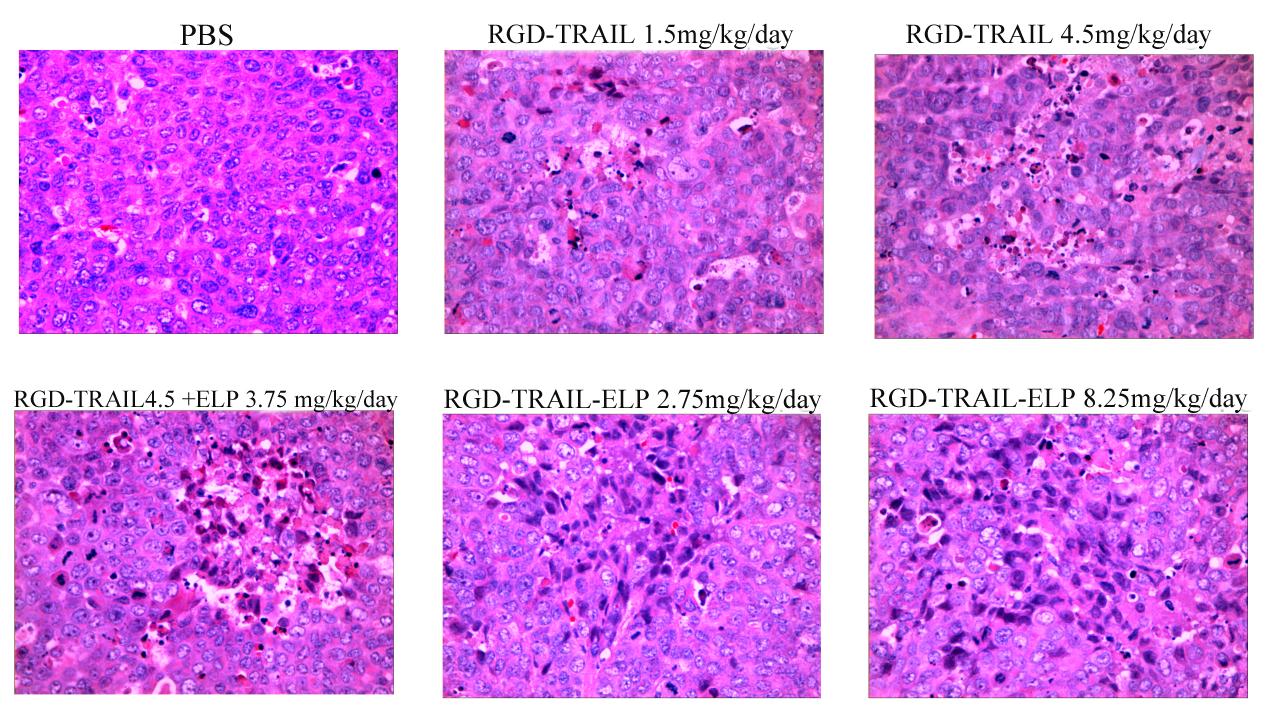
**Supplementary Fig. 7** Apoptosis assessment for RGD-TRAIL and TRAIL-ELP treatments using histological detection.


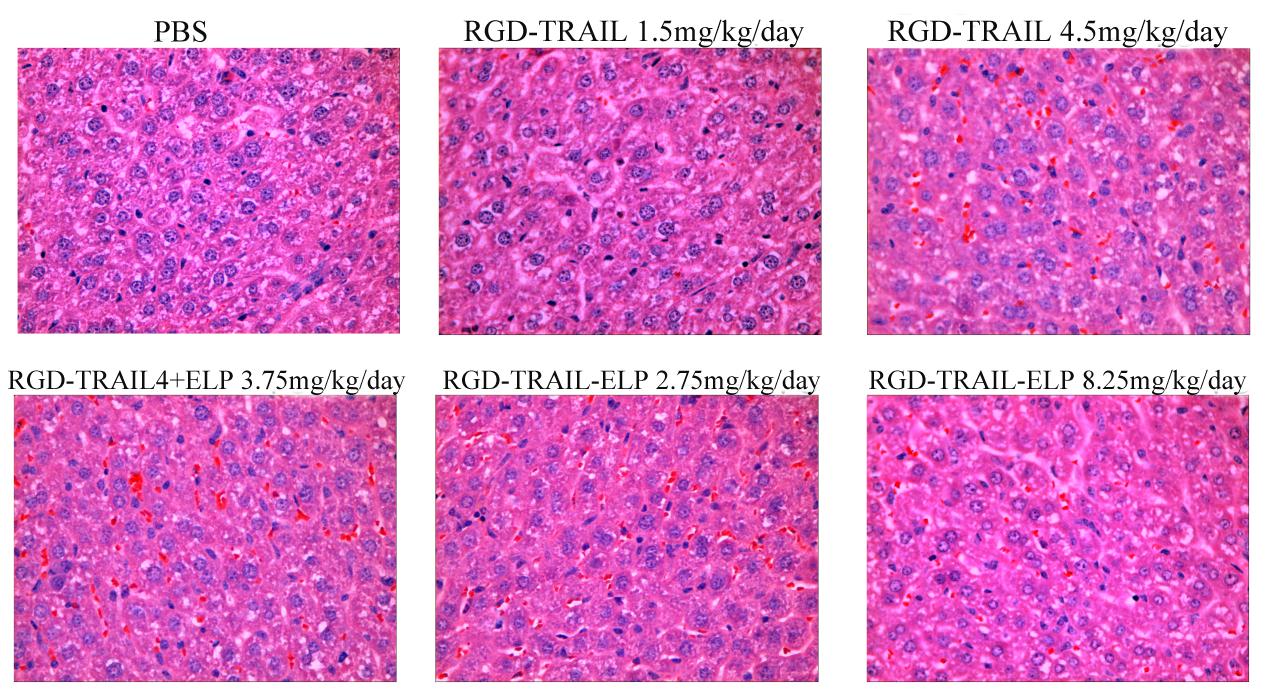


**Supplementary Fig. 8** cytotoxicity assessment for RGD-TRAIL and TRAIL-ELP treatments using histological detection.

**
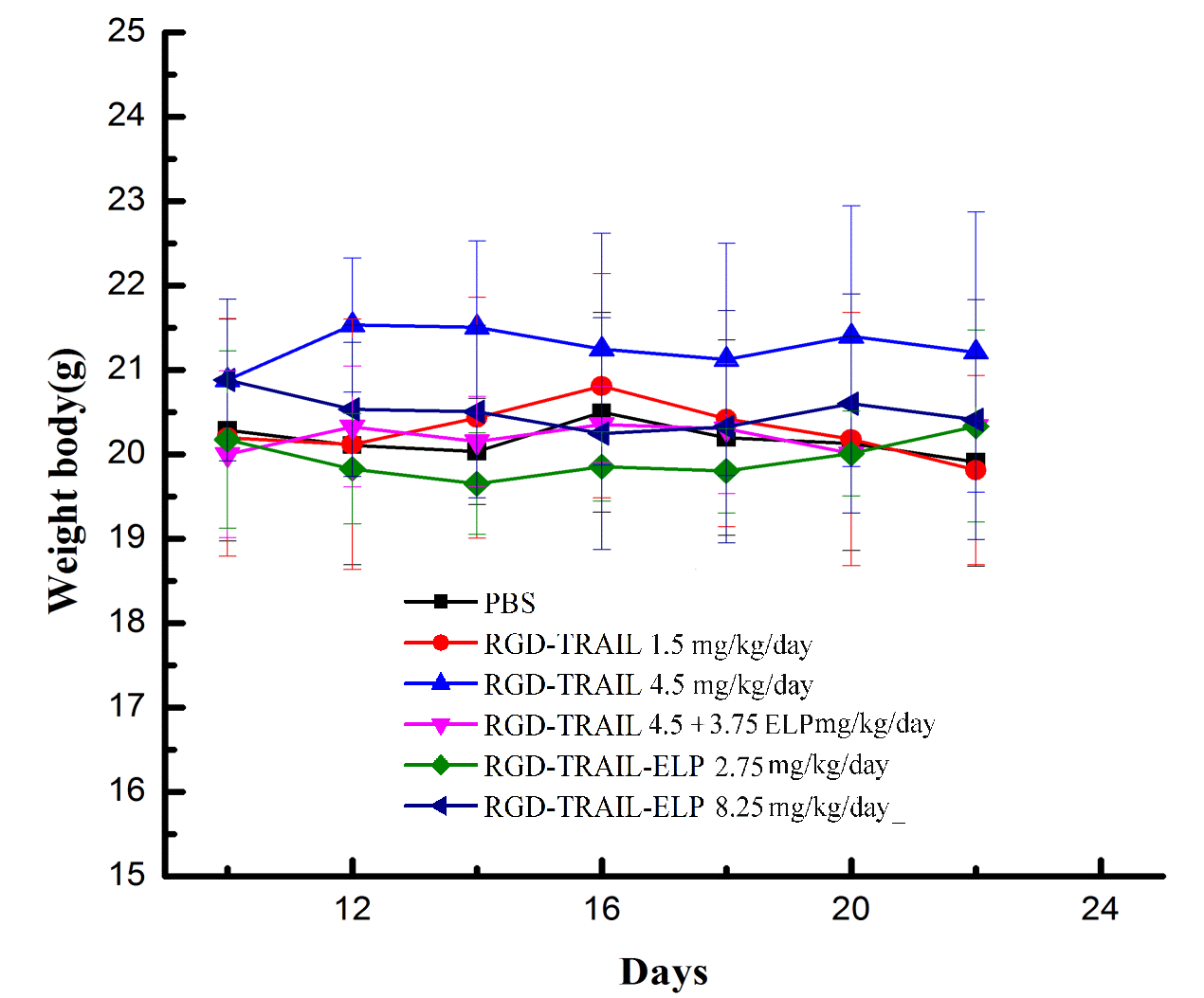
Supplementary Fig. 9** Body weight of each mouse in six groups was measured every other day. Results are expressed as mean ± S.D.


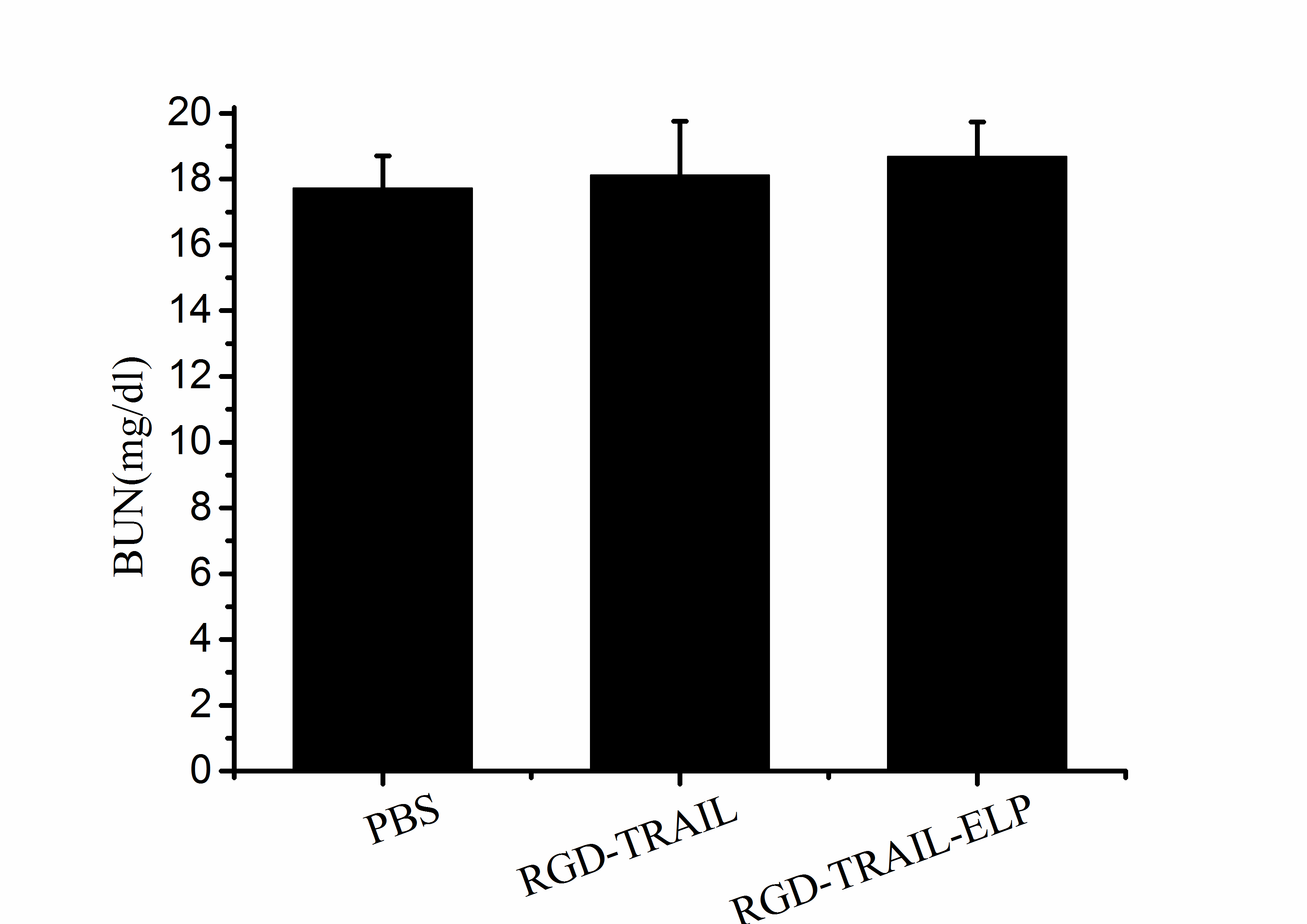


**Supplementary Fig. 10** Blood urea nitro-gen (BUN) analysis after administration


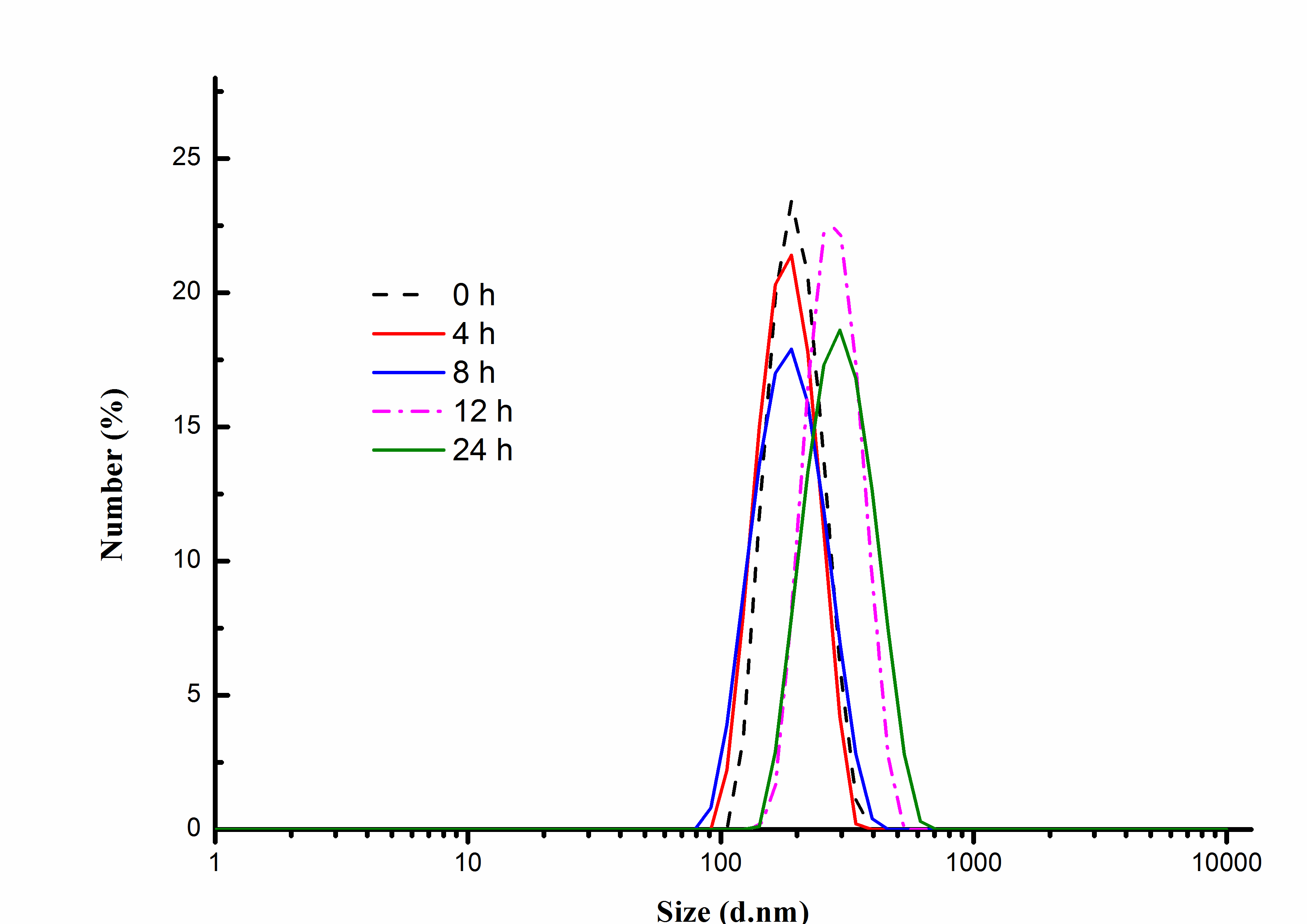


**Supplementary Fig. 11** Average diameter distribution of RGD-TRAIL-ELP over time.

**Table1:** Routine blood tests (expressed as mean ± SD)

| Items | PBS | RGD-TRAIL | RGD-TRAIL-ELP |
| --- | --- | --- | --- |
| RBC(×10^12^/L) | 8.82±0.8 | 7.97±1.12 | 7.82±1.58 |
| WBC(×10^9^/L) | 2.68±0.16 | 2.53±0.47 | 2.39±0.41 |
| PLT(×10^9^/L) | 1015.75±170.69 | 927.25±74.32 | 955.25±161.1 |
| HGB(g/L) | 151.89±16.12 | 138.95±29.88 | 140.13±43.2 |
